# Objectively measured physical activity in one-year-old children from a Brazilian cohort: levels, patterns and determinants

**DOI:** 10.1186/s12966-019-0895-1

**Published:** 2019-12-16

**Authors:** Luiza Isnardi Cardoso Ricardo, Inácio Crochemore M. da Silva, Otávio Amaral de Andrade Leão, Marlos Rodrigues Domingues, Fernando C. Wehrmeister

**Affiliations:** 1 Postgraduate Program in Epidemiology, Federal University of Pelotas, Marechal Deodoro, 1160 – 3rd Floor - Centro, Pelotas, RS Brazil; 2 Postgraduate Program in Physical Education, Federal University of Pelotas, Pelotas, Brazil

**Keywords:** Accelerometer, Children, Infant, Physical activity, Motor activity

## Abstract

**Background:**

The aim of this study is to describe objectively measured physical activity (PA) and its correlates in one-year-old children.

**Methods:**

The current study includes participants from the 2015 Pelotas (Brazil) birth cohort. At age one, PA was assessed in a 24-h protocol during 4 days with a wrist-attached accelerometer (ActiGraph, wGT3X-BT), from which two complete days of data were analyzed, with 5-s epochs.

**Results:**

A total of 2974 individuals provided valid accelerometry data. Infants able to walk independently spent on average 19 h per day below 50 m*g* of acceleration (including sleep time), and those who could not walk spent on average 21 h in this intensity category. Girls spent approximately 10 min more than boys below 50 m*g* daily in both walking status categories, and less activity than boys on higher intensity categories. Boys and infants whose mothers were more physically active during pregnancy presented more acceleration, regardless of walking status. Among infants who could walk by themselves, those with mothers with one to eight schooling years; adequate length-for-age (z-score); not attending daycare; and more physically active fathers also showed higher levels of acceleration.

**Conclusions:**

Our findings demonstrate higher levels of PA among boys and those children with higher maternal PA during pregnancy, regardless of walking status. Also, among infants able to independently walk, 1–8 years of maternal schooling, adequate length-for-age (z-score), no daycare attendance and higher paternal PA are positive correlates of objectively measured PA early in life.

## Background

The first years of life are considered critical for growth and development, with a great impact on present and future health. Behaviors acquired during childhood tend to persist in the future, pointing the early stages of life as an important period for the development of healthy behaviors [1, 2]. For example, there is evidence that physical activity (PA) at age 3 can predict PA in early adulthood [2]. Moreover, PA performed in the early years could improve health-related outcomes, such as adiposity, bone health, motor skills development, psychosocial health, cognitive development, and cardiometabolic health [3].

However, we know very little about infants’ and toddlers’ PA and its correlates [4]. In this age group, PA is composed by locomotion efforts and play activities, yet each child has different processes for motor skills acquisition and cognitive development, and those aspects are sensitive to environmental influences [5]. The existing evidence shows strong influences of family habits (parents and siblings) as the main correlates of children’s PA [6–9]. Also, evidence suggests child’s sex as a PA determinant among preschoolers, with boys being more active than girls [4, 7, 8]. Other topics are still underexplored in the literature, such as interaction with other children, daycare facilities’ structure and settings and other environmental factors [6, 10].

Therefore, knowledge regarding correlates and determinants of young children PA is still limited and controversial, mainly due to the mistaken notion that young children are naturally physically active [3]. Also, the literature points out the need for investigations on PA determinants in children from low- and middle- income countries, as well as research on determinants of PA in the early age ranges [7].

In this sense, one of the biggest challenges in determining infants’ PA is the measurement method. There is an increasing need to measure young children’s PA using reliable methods that contemplate the complexity of this behavior [11]. The use of accelerometers has been established as an important method for the measurement of PA, because it is an objective measure of body movement and theoretically less prone to bias [11]. Still, there are also limitations in using these devices. Some kind of movements are not well captured by the accelerometer [12]. The choice for placement of the device in young children can also be a challenge, as clothes or diapers may alter measurement when placed on the waist, or cause discomfort when placed on the ankle. Also, placing the accelerometer on the wrist could hamper crawling [13].

However, accelerometers have several advantages over other measurement methods, allowing the objective measurement of frequency, intensity and duration of activities for prolonged periods and with relatively small interference in the daily life of the participants [14]. Thus, the aim of the present study was to describe objectively measured overall PA and its correlates in one-year-old children belonging to the 2015 Pelotas (Brazil) birth cohort.

## Methods

### Study design

The current study includes participants from the 2015 Pelotas birth cohort. Pelotas is a city in southern Brazil with around 320,000 inhabitants. As in most Brazilian cities, socioeconomic inequalities are notable in Pelotas, expressed by an income Gini-Index of 0.42, as reported by the Brazilian Institute of Geography and Statistics (IBGE, 2003) [15]. The city’s economy is mostly based on high education and commerce. The present study was conducted between January and December, 2016, when the infants were one-year-old.

**Fig. 1:**
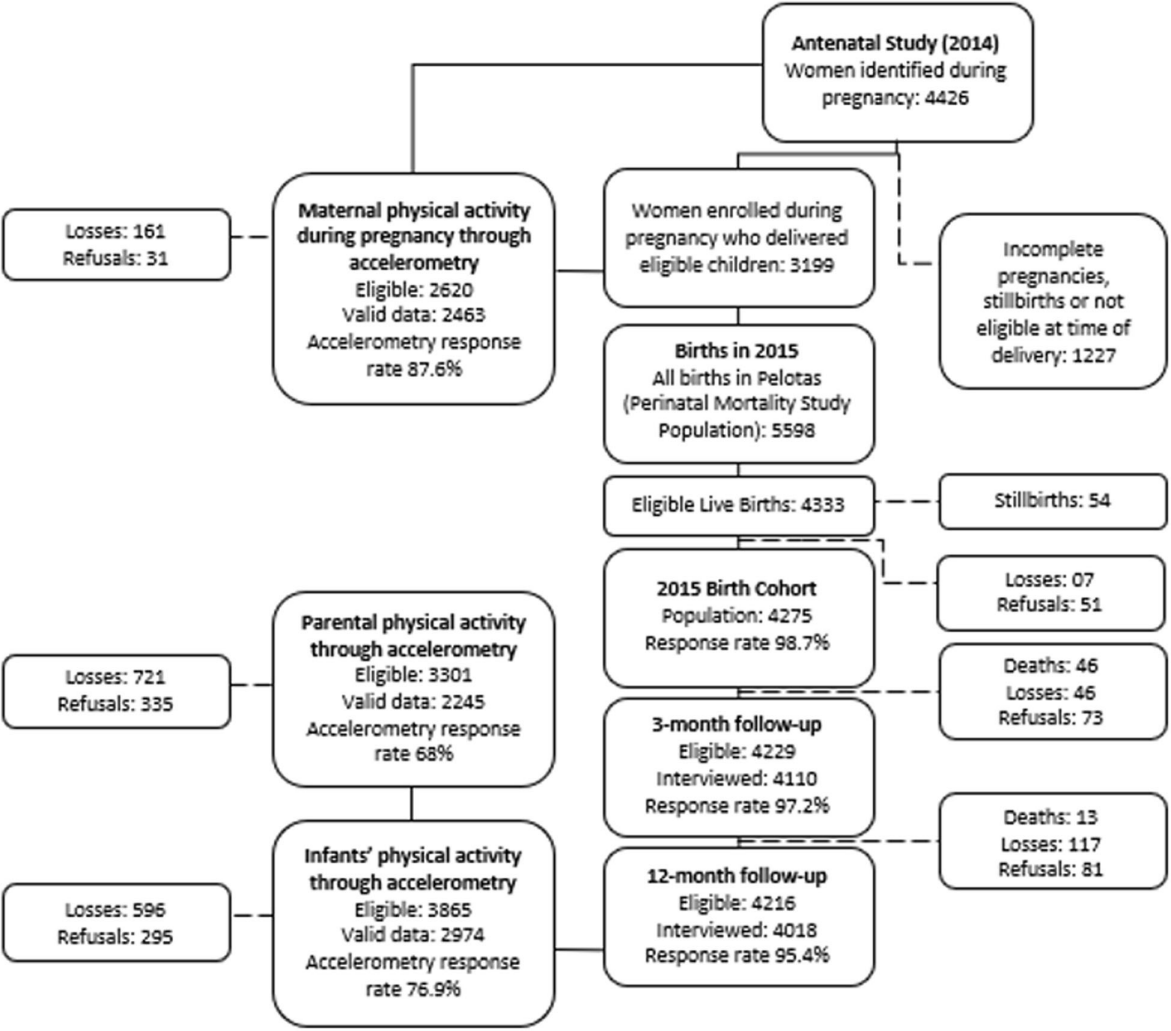
Study participant flowchart

To maintain consistency with the other cohort studies developed in the city [16–18], all hospital-delivered live-born infants between 1 January and 31 December 2015, whose mother lived in the urban area of the city, were eligible for inclusion in the study. Fig 1 shows a flowchart describing enrolment and participation. The 2015 birth cohort in Pelotas differed from the previous birth cohort studies by attempting to recruit pregnant women during antenatal care. The 123 health facilities and private clinics providing antenatal care in the city were visited or contacted weekly, between May 2014 and December 2015, to identify pregnant women expected to give birth during 2015. This parallel antenatal clinic study enrolled 73.8% of the mothers who subsequently delivered children included in the cohort. As shown in Fig. 1, 1227 woman identified in the antenatal study were not included in the 2015 birth cohort due to: incomplete pregnancies, stillbirths or not being eligible at the time of delivery.

In the one-year follow-up of the 2015 birth cohort, home visits were carried out, previously scheduled for the period between seven days before or after the child’s birthday. Parents/caregivers answered a questionnaire and anthropometric, clinical and biochemical measurements were performed. The one-year follow-up had a follow-up rate of 95.4% (Fig. 1). The study protocol was reviewed and approved by the School of Physical Education Ethics Committee at the Federal University of Pelotas (CAAE registration number: 26746414.5.0000.5313). More information regarding study design and measures of all early years of the 2015 Pelotas birth cohort follow-ups are available in the cohort profile [19].

### Accelerometry

Participants and their parents were invited and instructed to wear an accelerometer (ActiGraph, model wGT3X-BT, ActiGraph, USA), a waterproof device that measures acceleration in three axes (x, y, z) within a ± 6 *g* dynamic range. Accelerometers were set with a sampling frequency at 60hz, and a 5-s epoch was used. Data was expressed in milli-*g* (gravitational equivalent: 1000 m*g* = 1 *g* = 9.81 m/s^2^). For practical reasons and to increase compliance, PA was assessed using a 24-h protocol during four days: in the first day the interview was carried out and the accelerometer was placed; the accelerometer data collection started in the second day at midnight until the third day at midnight, and in the fourth day the device was removed. Participants were asked to remain with the device during the full data collection period.

The device was placed at the left wrist, using a disposable single-use bracelet to fix the accelerometer. The bracelet was made of waterproof vinyl (in white to avoid colorants), a safer material against contact dermatitis, widely used in the manufacture of surgical gloves [20]. All decisions regarding the bracelet material were established with a dermatologist specialized in the area. In the event of a dermatological complaint, a specialist was available to provide treatment. After the measurement period, accelerometers were collected by the research team at the participant’s home. This protocol was based on a pilot study (*N* = 90) which assessed children and caregivers’ preferences related to the place of accelerometer attachment and the minimum days necessary to represent day patterns of the children [13]. For parents, PA was assessed with the same device settings and data expression, and the accelerometer was placed in the non-dominant wrist.

Accelerometer data download and raw data .csv files extraction was performed in the Actilife 6.1 Software. Data were later analyzed with R-package GGIR (http://cran.r-project.org) in its continuous form, providing the average daily acceleration, expressing PA as a total volume of movement. The detailed signal processing scheme included the following steps: verification of sensor calibration error using local gravity as a reference [21]; detection of sustained abnormally high values and non-wear detection. The non-wear detection estimation was based on the standard deviation and value range from each accelerometer axis, classified in 15-min blocks and based on the characteristics of the 60-min window centered in these 15 min. A block was considered as non-wear time if, for at least two out of the three axes, the standard deviation was below 13.0 m*g* and the value range was below 50 m*g* [21].

Furthermore, GGIR package also calculates the vector magnitude of activity-related acceleration using the Euclidian Norm Minus One (ENMO to summarize three-dimensional raw data (from axis x, y, and z) (ENMO = ∑ | $\sqrt{x^2+{y}^2+{z}^2}$ – 1 g|). The data were further summarized when calculating the average values per 5-s-epochs and presented as the average acceleration originated by body movements per day (expressed in mg and as an estimate of overall PA volume). Further explanation for the raw data processing and analysis is available elsewhere [21, 22].

### Potential correlates of physical activity

The potential correlates were selected based on the literature regarding PA correlates of infants, toddlers and other age groups [4, 8, 23]. Therefore, potential correlates were defined as follows. Antenatal: mother’s PA accelerometer-assessed PA during pregnancy (average minutes per day spent in moderate-to-vigorous PA (MVPA), divided in tertiles). Perinatal: sex (female/male); low birth weight by hospital records defined as < 2500 g; Preterm birth, defined as < 37 weeks of gestation according to the last menstrual period and ultrasound; Maternal age (< 20; 20–29; 30–39; ≥40 years); Assets index, generated by a standardized socioeconomic questionnaire [24], including questions on household assets, the presence of a maid, and education level of the head of the household (categorized in quintiles based on a principal component analysis); Maternal and Paternal schooling (0; 1–8; 9–11; ≥12 years). Twelve months follow-up: Number of siblings (0; 1; ≥2); Daycare attendance (yes; no); Contact with other children (yes; no), based on the presence of other children in the daily care environment; Length-for-age (<− 2 SD; ≥ − 2 SD in z-score); Paternal PA (average minutes per day spent in MVPA, divided in tertiles); and child motor development. For the motor development measure the Oxford Neurodevelopment Assessment (OX-NDA) was used, which also assessed the domains of language, cognitive, executive, attention, socio emotional reactivity and positive affection [25].

Parents’ MVPA was objectively measured using Actigraph GT3x + wrist-worn accelerometer, for 24 h, during seven consecutive days. The data was analyzed in its raw form (m*g*), processed and filtered similar to the infants’ accelerometry data. The time spent in MVPA (> 100 m*g*) [26] was analyzed without bout criteria and presented in tertiles for analysis purposes.

At the one-year follow-up children were weighted on their mothers’ laps using SECA 803 scales (SECA, Germany) with 100 *g* precision; the mother then handed the child to someone else and her weight was obtained in the same scale. The child’s weight was provided automatically by the scale as the difference between the two weights. Infants had their length measured using a SANNY ES2000 portable anthropometer (SANNY, Brazil) with 5 mm precision and were weighted using a portable electronic scale with 10 g precision. The BMI for age z-score was categorized using the WHO protocols and cut-off points based on the growth curves [27].

### Statistical analysis

All the analyses were carried out with the statistical package Stata 12.1 (StataCorp., 2011), stratifying for infants’ walking status (capable or incapable of independently walking). Descriptive analyses were performed based on valid data from all participants who provided at least one full day of measurement according to the protocol. Based on a previous protocol study, one full day still gives a good estimate of infant’s movement [13], and infants with less than two measurement days represented 0.57% of the analytical sample (*N* = 17). Comparison between compliant and non-compliant individuals were performed using chi-squared tests. Outcome distribution were checked graphically using a histogram. Multiple linear regressions using multiplicative terms were carried out to examine potential interactions between variables.

Unadjusted and adjusted analyses were performed using linear regressions. All variables with more than two categories were dummy-coded with a reference group. However, to better understand the results, the marginal means for each category were presented instead of the regression coefficients. All potential correlates were entered in the model at the same time, therefore all adjusted analysis considered the following variables: maternal age, assets index, maternal and paternal schooling, maternal PA during pregnancy, low birth weight, infants’ sex, prematurity, length-for-age, number of siblings, daycare attendance, contact with other children, motor development and paternal PA.

Multicollinearity was evaluated through correlation matrixes of all the included variables, and although there is moderate correlation between (a) assets index and maternal schooling (r = 0.60); (b) assets index and paternal schooling (r = 0.58), this does not impact PA estimates, since the mean variance inflation factors for the independent variables were also evaluated, and no indication of estimation bias were found. Statistical significance was set at 5, and 95% confidence intervals are provided.

## Results

A total of 4018 participants and their mothers/caregivers were interviewed at the one-year follow-up. Of those, 153 were considered not eligible for the accelerometry data collection because they were not living in the urban area of Pelotas. Also, 596 individuals were considered losses and 295 refused to participate. Therefore, 2974 individuals provided valid accelerometry data, comprising 74% of the participants interviewed in the Cohort study (Table 1) and 76.9% of the sample eligible for accelerometer data collection (Fig. 1). Our sample presented higher proportion of males (*p* = 0.008) and individuals from lower socioeconomic status (*p* < 0.001), in comparison with the remaining cohort members followed-up. There were no statistical differences between compliant and non-complaint individuals regarding preterm birth, low birth weight and maternal age.

**Table 1:** Characteristics of the one-year-old participants. The 2015 Pelotas Birth Cohort, Brazil

Characteristics	Accelerometry data^*^	No accelerometry data^**^	
	n (%)	n (%)	*p*-value (x^2^ test)
Sex			0.008
Female	1424 (47.9)	550 (52.7)	
Male	1550 (52.1)	494 (47.3)	
Low birthweight			0.109
Yes	267 (9.0)	111 (10.7)	
No	2707 (91.0)	930 (89.3)	
Preterm birth			0.490
Yes	442 (14.9)	146 (14.0)	
No	2532 (85.1)	898 (86.0)	
Maternal age (years)			0.065
< 20	446 (15.0)	139 (13.3)	
20–29	1425 (47.9)	470 (45.0)	
30–39	1020 (34.3)	401 (38.4)	
≥ 40	82 (2.8)	34 (3.3)	
Assets index (quintiles)			
1 (poorest)	590 (20.5)	171 (17.0)	< 0.001
2	606 (21.1)	177 (17.6)	
3	599 (20.8)	188 (18.7)	
4	573 (19.9)	209 (20.7)	
5 (richest)	507 (17.6)	263 (26.1)	
Overall	2974 (100)	1044 (100)	

^*^Number of observations vary due to missing values

^**^Participants with no accelerometry valid data

^ǂ^ Includes 59 deaths

**Fig. 2:**
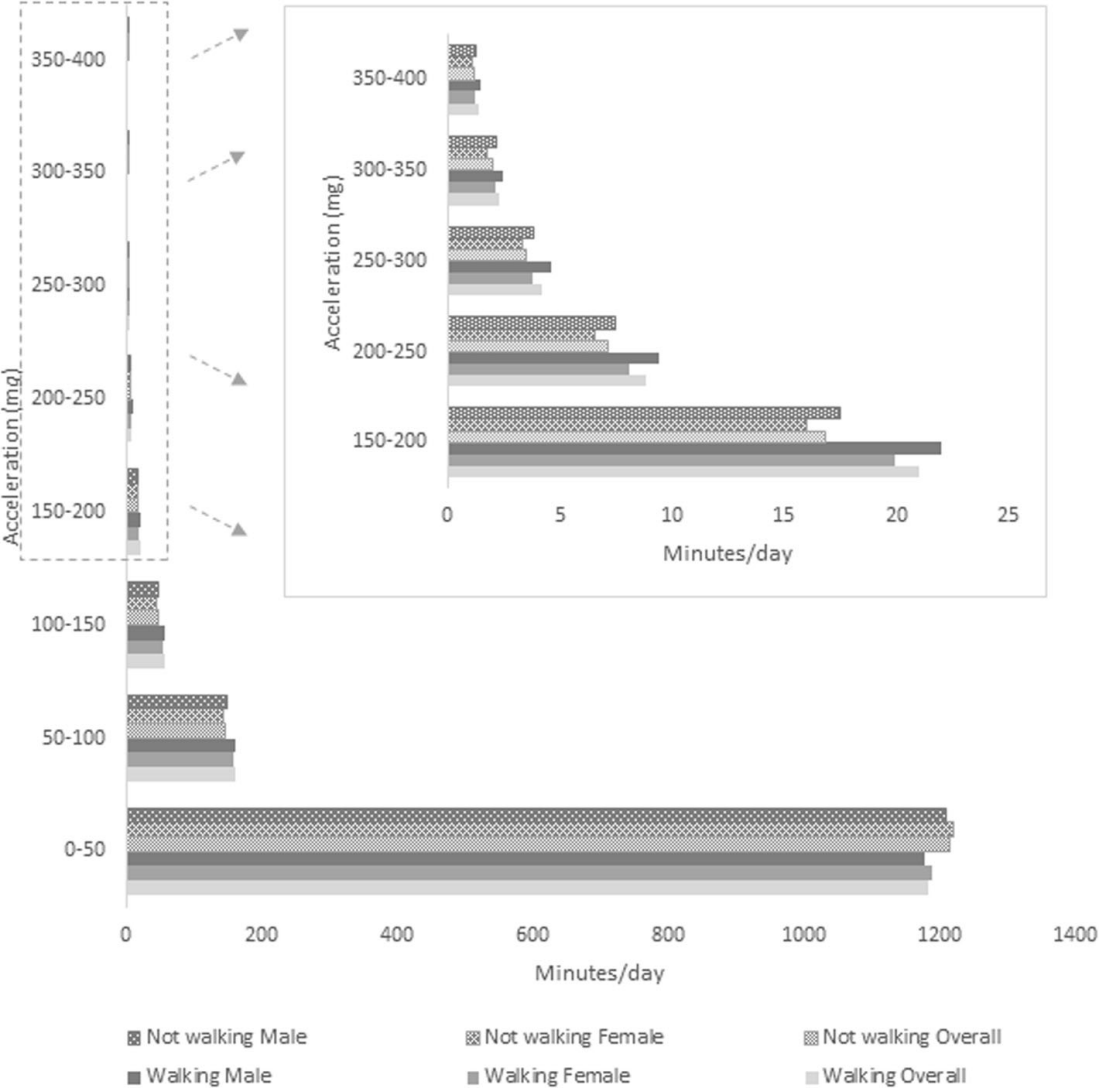
Acceleration distribution of time spent in accelerometry intervals of 50-m*g* among one-year-old infants. 2015 Pelotas birth cohort, RS - Brazil

Figure 2 shows the acceleration distribution of infants’ time spent in accelerometry intervals of 50-m*g*, representing intensity categories, stratified by walking status. Infants able to walk independently spent on average 19 h per day below 50 m*g* of acceleration, and those who could not walk spent on average 21 h in this intensity category. Girls spent approximately 10 min more than boys below 50 m*g* daily in both walking status categories (walking girls: 1187.2, walking boys: 1178; non-walking girls: 1220, non-walking boys: 1208.7). In higher intensity categories, from 50 m*g* onwards, boys presented more activity than girls, and this difference is more pronounced among infants that could walk independently.

Crude and adjusted association between PA (m*g*) and antenatal, perinatal and one-year characteristics, obtained by linear regression are presented in Tables 2 and 3. In the crude analysis, infants’ PA was associated with maternal PA during pregnancy, sex, daycare attendance, motor development and paternal PA. When stratifying for walking status, PA was associated with maternal schooling, daycare attendance and contact with other children, only for infants that could walk independently. However, maternal PA during pregnancy, sex and paternal PA were associated with infants’ PA regardless of walking status.

**Table 2:** Crude association between physical activity (m*g*) and antenatal, perinatal and one-year characteristics among one-year-old infants. 2015 Pelotas birth cohort, RS - Brazil

	Acceleration (m*g*) ^¥^					
	Overall		Walking		Non walking	
Characteristics	Mean (95% CI)	*p*-value^#^	Mean (95% CI)	*p*-value^#^	Mean (95% CI)	*p*-value^#^
Maternal age (years)		0.053		0.277		0.333
< 20	27.0 (26.4; 27.6)		28.4 (27.5; 29.3)		25.8 (25.0; 26.6)	
20–29	26.3 (25.9; 26.6)		27.7 (27.2; 28.2)		25.0 (24.5; 25.4)	
30–39	26.3 (25.9; 26.7)		27.6 (27.0; 28.2)		25.2 (24.7; 25.7)	
≥ 40	25.5 (24.1; 26.9)		27.9 (25.6; 30.2)		24.3 (22.5; 26.0)	
Assets index (quintiles)		0.814		0.108		0.625
1 (poorest)	26.1 (25.6; 26.6)		27.7 (26.9; 28.5)		24.9 (24.3; 25.6)	
2	26.5 (26.0; 27.0)		28.4 (27.6; 29.3)		25.0 (24.3; 25.6)	
3	26.8 (26.3; 27.3)		28.2 (27.4; 29.0)		25.6 (24.9; 26.3)	
4	26.2 (25.7; 26.7)		27.0 (26.3; 27.8)		25.5 (24.8; 26.2)	
5 (richest)	26.1 (25.6; 26.7)		27.3 (26.4; 28.2)		24.9 (24.2; 25.6)	
Maternal schooling (years)		0.286		0.005		0.883
0	26.6 (25.8; 27.4)		29.0 (27.7; 30.4)		25.2 (24.3; 26.1)	
1–8	26.5 (26.0; 26.9)		28.1 (27.4; 28.8)		25.0 (24.4; 25.6)	
9–11	26.4 (26.0; 26.8)		27.8 (28.2; 28.4)		25.2 (24.7; 25.7)	
≥12	26.2 (25.8; 26.6)		27.1 (26.5; 27.8)		25.2 (24.6; 25.8)	
Paternal schooling (years)		0.974		0.163		0.940
0	26.0 (25.3; 26.6)		28.1 (27.0; 29.2)		24.8 (24.0; 25.6)	
1–8	26.6 (26.2; 27.1)		27.7 (27.1; 28.4)		25.6 (25.0; 26.2)	
9–11	26.4 (25.9; 26.8)		28.1 (27.4; 28.8)		25.0 (24.4; 25.5)	
≥ 12	26.2 (25.7; 26.7)		27.0 (26.3; 27.8)		25.2 (24.5; 25.9)	
Maternal PA during pregnancy (tertiles)^ǂ^		< 0.001		< 0.001		0.050
1 (lowest)	25.3 (24.8; 25.9)		25.9 (25.1; 26.8)		24.7 (24.0; 25.4)	
2	26.7 (26.2; 27.3)		28.2 (27.4; 29.1)		25.3 (24.6; 26.0)	
3 (highest)	27.5 (26.9; 28.0)		28.9 (28.1; 29.7)		25.7 (25.0; 26.5)	
Low birthweight		0.703		0.399		0.831
Yes	26.4 (26.2; 26.6)		28.4 (26.9; 29.8)		25.3 (24.4; 26.1)	
No	26.2 (25.5; 27.0)		27.7 (27.4; 28.1)		25.1 (24.8; 25.5)	
Infant’s sex		< 0.001		0.0015		< 0.001
Female	25.8 (25.4; 26.1)		27.2 (26.6; 27.7)		24.5 (24.1; 25.0)	
Male	26.7 (26.6; 27.3)		28.3 (27.8; 28.8)		25.7 (25.3; 26.1)	
Preterm birth		0.561		0.680		0.736
Yes	26.2 (25.6; 26.8)		28.0 (26.9; 29.0)		25.3 (24.6; 26.0)	
No	26.4 (25.6; 26.8)		27.8 (27.4; 28.1)		25.1 (24.8; 25.5)	
Length-for-age (z-score)		0.062		0.278		0.724
< −2 sd	25.3 (24.1; 26.4)		26.7 (24.7; 28.7)		24.9 (23.5; 26.3)	
≥ − 2 sd	26.4 (26.1; 26.6)		27.8 (27.4; 28.2)		25.2 (24.8; 25.5)	
Number of siblings		0.111		0.125		0.009
0	26.7 (26.3; 27.0)		27.6 (27.1; 28.1)		25.7 (25.2; 26.1)	
1	26.0 (25.6; 26.4)		27.6 (26.9; 28.2)		24.7 (24.2; 25.3)	
≥ 2	26.3 (25.8; 26.8)		28.6 (27.7; 29.4)		24.7 (24.0; 25.4)	
Daycare attendance		0.030		0.019		0.567
No	26.5 (26.2; 26.7)		27.9 (27.6; 28.3)		25.2 (24.9; 25.5)	
Public	25.1 (23.8; 26.4)		27.2 (25.2; 29.3)		23.5 (21.8; 25.1)	
Private	25.8 (25.0; 26.5)		26.5 (25.3; 27.7)		25.2 (24.2; 26.2)	
Contact with other children		0.095		0.023		0.771
Yes	25.9 (25.3; 26.5)		26.7 (25.8; 27.7)		25.3 (24.5; 26.1)	
No	26.5 (26.2; 26.7)		27.9 (27.6; 28.3)		25.1 (24.8; 25.5)	
Motor development (tertiles)		< 0.001		0.344		0.179
1 (lowest)	25.7 (25.3; 26.1)		27.8 (26.8; 28.9)		25.3 (24.9; 25.7)	
2	26.1 (25.7; 26.5)		27.3 (26.7; 28.0)		25.0 (24.4; 25.5)	
3 (highest)	27.5 (27.0; 27.9)		28.0 (27.5; 28.5)		24.7 (23.8; 25.7)	
Paternal PA (tertiles)		< 0.001		0.0025		0.003
1 (lowest)	25.5 (25.0; 26.0)		26.5 (25.8; 27.3)		24.5 (23.8; 25.2)	
2	26.7 (26.2; 27.2)		28.0 (27.3; 28.7)		25.4 (24.7; 26.1)	
3 (highest)	27.0 (26.5; 27.4)		28.3 (27.4; 28.9)		25.9 (25.2; 26.5)	

^¥^Marginal means obtained through linear regression ^#^ Heterogeneity *p*-value ^ǂ^Variable with highest missing values (*n* = 1463)

**Table 3:** Adjusted association between physical activity (m*g*) and antenatal, perinatal and one-year characteristics among one-year-old infants. 2015 Pelotas birth cohort, RS - Brazil

	Acceleration (m*g*) ^¥^					
	Overall		Walking		Non walking	
Characteristics	Mean (95% CI)	*p*-value^#^	Mean (95% CI)	*p*-value^#^	Mean (95% CI)	*p*-value^#^
Maternal age (years)		0.465		0.656		0.320
< 20	26.9 (25.5; 28.4)		28.4 (26.2; 30.5)		25.4 (23.5; 27.3)	
20–29	26.2 (25.6; 26.8)		27.5 (26.7; 28.3)		24.9 (24.1; 25.8)	
30–39	26.8 (26.1; 27.5)		28.0 (26.8; 29.1)		25.9 (25.0; 26.7)	
≥ 40	27.4 (25.1; 29.6)		29.4 (25.8; 32.9)		25.8 (23.0; 28.6)	
Assets index (quintiles)		0.116		0.351		0.191
1 (poorest)	25.1 (23.9; 26.3)		26.1 (24.3; 27.8)		24.1 (22.7; 25.9)	
2	26.5 (25.5; 27.5)		28.5 (27.0; 30.1)		24.9 (23.7; 26.1)	
3	27.1 (26.2; 27.9)		28.1 (26.8; 29.4)		25.8 (24.7; 26.9)	
4	27.0 (26.1; 27.8)		28.1 (26.9; 29.2)		25.9 (24.8; 27.0)	
5 (richest)	26.6 (25.6; 27.5)		27.7 (26.1; 29.2)		25.6 (24.3; 26.8)	
Maternal schooling (years)		0.047		0.028		0.744
0	27.2 (25.4; 29.1)		28.7 (25.9; 31.6)		25.5 (23.1; 27.9)	
1–8	27.5 (26.4; 28.5)		29.5 (28.0; 31.1)		25.4 (24.0; 26.9)	
9–11	26.7 (26.1; 27.3)		27.8 (26.8; 28.7)		25.6 (24.8; 26.5)	
≥12	25.8 (25.1; 26.6)		26.8 (25.6; 27.9)		25.1 (24.1; 26.1)	
Paternal schooling (years)		0.785		0.411		0.726
0	26.7 (25.3; 28.1)		28.1 (25.9; 30.3)		25.2 (23.4; 27.0)	
1–8	26.2 (25.4; 27.1)		26.7 (25.4; 27.9)		25.8 (24.7; 27.0)	
9–11	26.7 (26.1; 27.4)		28.3 (37.3; 29.3)		25.3 (24.4; 26.2)	
≥ 12	26.6 (25.7; 27.5)		28.1 (26.7; 29.4)		25.2 (24.0; 26.4)	
Maternal PA during pregnancy (tertiles)^ǂ^		< 0.001		< 0.001		0.013
1 (lowest)	25.1 (24.4; 25.8)		25.9 (24.9; 27.0)		24.4 (23.5; 25.3)	
2	27.2 (26.5; 27.8)		28.6 (27.6; 29.6)		25.8 (24.9; 26.6)	
3 (highest)	27.4 (26.7; 28.1)		28.8 (27.8; 29.8)		26.1 (25.1; 27.0)	
Low birthweight		0.733		0.932		0.783
Yes	26.3 (24.5; 28;0)		27.8 (27.2; 28.4)		25.2 (23.1; 27.2)	
No	26.6 (26.2; 27.0)		27.6 (24.4; 30.9)		25.4 (24.9; 26.0)	
Infant’s sex		< 0.001		0.003		0.004
Female	25.6 (25.0; 26.2)		26.8 (25.9; 27.7)		24.6 (23.8; 25.3)	
Male	27.4 (26.8; 27.9)		28.6 (27.8; 29.4)		26.1 (25.4; 26.8)	
Preterm birth		0.554		0.749		0.747
Yes	26.2 (25.0; 27.4)		27.5 (25.4; 29.5)		25.2 (23.8; 26.6)	
No	26.6 (26.2; 27.0)		27.8 (27.1; 28.4)		25.4 (24.9; 26.0)	
Length-for-age (z-score)		0.085		0.031		0.933
< −2 sd	24.7 (22.6; 26.8)		23.8 (20.1; 27.5)		25.3 (22.8; 27.8)	
≥ − 2 sd	26.6 (26.2; 27.0)		27.9 (27.3; 28.5)		25.4 (24.9; 25.9)	
Number of siblings		0.464		0.562		0.620
0	26.9 (26.3; 27.4)		28.1 (27.2; 29.0)		25.7 (24.9; 26.5)	
1	26.1 (25.4; 26.7)		27.4 (26.3; 28.4)		24.9 (24.0; 25.8)	
≥ 2	26.7 (25.6; 27.8)		27.7 (26.0; 29.4)		25.6 (24.1; 27.2)	
Daycare attendance		0.148		0.046		0.930
No	26.8 (26.3; 27.3)		28.2 (27.5; 28.9)		25.6 (25.0; 26.2)	
Public	24.5 (22.1; 26.8)		26.8 (23.1; 30.5)		22.5 (19.5; 25.4)	
Private	25.3 (23.5; 27.0)		25.2 (22.6; 27.8)		25.4 (23.1; 27.6)	
Contact with other children		0.430		0.811		0.524
Yes	26.0 (24.7; 27.4)		27.5 (25.3; 29.7)		24.9 (23.2; 26.6)	
No	26.7 (26.2; 27.1)		27.8 (27.1; 28.6)		25.5 (24.9; 26.1)	
Motor development (tertiles)		0.016		0.619		0.134
1 (lowest)	26.0 (25.3; 26.7)		27.5 (25.8; 29.2)		25.6 (25.0; 26.3)	
2	26.5 (25.8; 27.3)		27.6 (26.5; 28.8)		25.4 (24.5; 26.4)	
3 (highest)	27.2 (26.5; 27.8)		27.9 (27.1; 28.7)		24.2 (22.8; 25.7)	
Paternal PA (tertiles)		0.003		0.006		0.226
1 (lowest)	25.6 (24.9; 26.4)		26.5 (25.4; 27.6)		24.9 (24.0; 25.8)	
2	26.8 (26.1; 27.5)		28.0 (27.0; 28.9)		25.7 (24.8; 26.6)	
3 (highest)	27.2 (26.5; 28.0)		28.8 (27.7; 29.9)		25.7 (24.7; 26.7)	

^¥^ Adjusted marginal means obtained through linear regression ^#^ Heterogeneity p-value ^ǂ^Variable with highest missing values (n = 1463)

When adjusting for all the variables, maternal schooling and paternal PA remained associated with PA for the overall sample (*p* = 0.047; *p* = 0.003, respectively) and for infants that could walk (*p* = 0.028; *p* = 0.006, respectively). Length-for-age (z-score), daycare attendance showed significant association only for infants that could walk (*p* = 0.031; *p* = 0.046, respectively). Motor development was associated with PA only for the overall sample (*p* = 0.016), losing significance when stratified by walking status. However maternal PA during pregnancy and sex remained associated with infant’s acceleration after adjustment for the remaining variables and stratification for walking status.

Infants whose mothers were in the 3rd tertile of gestational PA presented body acceleration on average 2.34 m*g* (overall: mean 1st tertile: 25.1, 95% CI 24.4–25.8; mean 3rd tertile: 27.4,, 95% CI 26.7–28.1; *p* < 0.001) higher than the 1st tertile, regardless of walking status (Table 3). Female infants presented less acceleration than males (overall girls: mean 25.6, 95% CI 25.0–26.2; boys: mean 27.4, 95% CI 26.8–27.9; *p* < 0.001), and the association remained regardless of walking status (walking girls: mean 26.8, 95% CI 25.9–27.7; boys: mean 28.6, 95% CI 27.8–29.4; *p* < 0.001; non-walking girls: mean 24.6, 95% CI 23.8–25.3; boys mean 24.6, 95% CI 23.8–25.3).

Among infants that could walk, those with mothers with one to eight schooling years (mean 29.5 m*g*, 95% CI 28.0–31.1) had higher acceleration when compared with higher maternal schooling categories (9-11 yr: mean 27.8 m*g*, 95% CI 26.8–28.7; ≥12 yr: 26.8 m*g*, 95% CI 25.6–27.9) but no statistical difference were found with lower maternal schooling (mean 28.7 m*g,* 95% CI 25.9–31.6). Infants with adequate length-for-age (z-score) presented on average 4.1 m*g* more PA. Infants not attending daycare (mean 28.2 m*g*, 95% CI 27.5–28.9) presented more PA then those attending public (26.8 m*g*, 95% CI 23.1–30.5) or private (25.2 m*g*, 95% CI 22.6–27.8) daycare facilities. Also, infants whose fathers were in the 3rd tertile of paternal PA presented, on average, 2.3 m*g* more acceleration than those in the 1st tertile of paternal PA (*p* = 0.006) (Table 3).

## Discussion

This study presents objectively measured PA data and associations with potential determinants of PA in almost 3000 infants belonging to a population-based cohort study in southern Brazil. To the best of our knowledge this is the first study to provide population-based estimates of PA obtained by wrist-attached raw accelerometers among infants.

Our findings showed higher maternal PA during pregnancy and male sex are positively associated with infants’ PA, regardless of walking status. In addition, among infants able to independently walk, middle maternal schooling, adequate length-for-age (z-score), no daycare attendance and higher paternal PA are positive correlates of infants objectively measured PA. These results were consistent with evidence from studies with older children. The “Generation R” study (Netherlands), conducted with two-year-old children, aimed at identifying determinants of PA measured by accelerometry. The results showed that PA was higher among boys; older children; and those with more than two brothers; however, PA was lower during the winter. These factors were associated with at least one component of the PA in the sample studied (total PA, mild, moderate, vigorous, moderate to vigorous and counts per minute) [4]. Also, considering the age group up to six years, a recent review study provides evidence about the main determinants and factors associated with PA. According to this study, the main determinants and factors associated with children’s PA, regardless of the method of measurement, were factors such as: sex (boys are more active), parental PA, parental social support, time spent outdoors and time playing with parents [8].

To better understand PA estimates in the present study some conceptual issues need to be addressed. Firstly, the PA captured by the accelerometer has different meaning from infants to other age groups, being basically composed by active play and locomotion efforts. However, the interpretation of the acceleration and the magnitude of the differences found is complex, even among older age groups, the translation of these data for real life activities is only possible through calibration studies. In this sense, it is important to discuss if there is a need to classify infants and toddlers regarding PA intensity thresholds, since descriptive analysis using overall activity could minimize excessive arbitrariness and may better represent the behavior. Second, we present PA as raw data (m*g*), which differs from traditional accelerometry analysis. Unlike count-based estimates (a specific count metrics by each accelerometer manufacturer), raw data analyzes allow researchers to control data processing, since it is possible to select which attributes will be run by the algorithm. Also, raw data analyzes could enhance comparability between different accelerometer brands, since the data filtering process applied by the different brands can affect the results in various ways [21, 28].

Our findings showed that most of children daily time was spent in low intensity activities, such as sleep time, sedentary behavior and/or sitting and reclined activities with minimum upper limb movements, for example. Furthermore, the present study considered total raw acceleration, and therefore included sleep time in the average daily acceleration. Beyond night sleep, daytime naps are common at this age, lasting up to 3 h in duration approximately [29]. The recommendations for one-year-old infants is to sleep for 11–14 h during night time and more 3 h of daily naps [30]. At this age, the home environment and daycare facilities are the places where children spend most of their time, having a greater potential for actions to encourage development, as well as the beginning of their PA behavior [31, 32]. To better understand the patterns of PA and the translation of accelerometer measures to infants’ activities, calibration studies would be needed.

Our study found that boys were more physically active than girls. This pattern seems to be persistent in the literature, in agreement with the knowledge already established for other age groups. A European study found that both counts per minute and moderate-to-vigorous PA were higher in boys, whereas sedentary time was higher for girls, when analyzing children from 2 to 10 years old [33]. Similarly, a Canadian study showed that female adolescents (12 years old) spent significantly more time in sedentary activities and less time in MVPA when compared to males. These differences could be largely explained by socio-cultural aspects, such as incentive to active play and environmental exploration, usually more common among boys. In this sense, girls receive less favorable influences by socio-ecological factors at the individual, family, school and environmental levels [34]. It is important to highlight that these factors are changeable, and could be improved by giving appropriate instructions to parents and teachers about the benefits of PA in infancy.

Our results showed that an adequate length-for-age z-score was associated with higher infant PA. This score represents not only growth standards but also links between physical growth and motor development in infancy [27]. Infants and toddlers with higher scores probably had parents with healthy practices such as breastfeeding and not smoking during and after pregnancy [27].

Elementary-level maternal education (between 1 and 8 years of education) was associated with higher infant PA in the present study. Maternal education is commonly associated with children’s PA in the literature, representing a proxy of socioeconomic status [35, 36]. Among infants, we believe maternal education can also represent time spent with the child, where infants with highly educated mothers spend more time in daycare while those with slightly less educated mothers spend more time at home. Also, infants attending childcare presented less PA than those who do not. The childcare methods and organization can act as a facilitator or a barrier to children’s PA. Some studies have shown that children spent little time in physical activities in school or childcare time [37, 38], and those who attend childcare may spent more time in light intensity activities, sedentary behavior or sleeping [31, 39].

Our results showed that the higher the infants’ motor development, the higher the PA level, however, when stratifying for walking status the association was no longer significant. This may be because walking is the major proxy of motor development at this age group. One review about health indicators in children (0–4 years) showed that high levels of PA were associated with cognitive and motor skill development [3]. The literature indicates that children with better motor proficiency tend to move more when compared with less developed children [40], pointing the motor development as an important PA determinant in this age. This relationship could be considered bidirectional because motor development and PA both influence each other, and since both variables were evaluated at the same timeframe, causality cannot be inferred. However, actions to improve both PA and motor development could be considered complementary, such as incentive to active play and environment exploration.

Paternal and children’s PA collected simultaneously was strongly associated with infants’ behavior in the present study. Also, maternal PA during pregnancy was positively associated with higher infant acceleration regardless of walking status. Literature regarding gestational PA usually focuses on maternal and newborn effects, and although inconsistent, evidence shows that gestational PA can positively influence child’s health [41]. Because the area is still recent, no studies evaluating effects of gestational PA and infants’ PA were found.

Meanwhile, parental PA appears to be a consistent determinant of child behavior. A review study showed that, among children aged 2–5 years, active parents tend to positively influence their children [8]. In addition, active parents tend to encourage more PA of children up to 5 years of age, positively impacting PA during adolescence [9]. Thus, parental PA could affect infants’ PA through behavioral aspects, such as incentive to movement and freedom to play. On the other hand, despite the expected behavioral influence, gestational PA could have a physiological meaning on the infants’ organism, consequently differing from the paternal PA association encountered in the present study. Therefore, further research is needed to better explain the possible effects of gestational PA on child behavior.

A few methodological considerations when measuring infants’ PA are worth mentioning. Based on a previous pilot study, the wrist was the placement with better compliance and comfort compared to the ankle, and the PA estimates also had greater reliability coefficients with lesser periods of measurement. However, a few mothers reported difficulties for infants that were still crawling, because the device may cause discomfort [13]. Furthermore, regarding walking status, at one year of age it is possible for a healthy infant not to be capable of walking, depending on several processes and stimuli that may or may not have occurred in this period [40]. Therefore, it is crucial to stratify the PA analysis for walking status when analyzing data from one-year-old infants.

Some limitations should be considered in this study. Our analytical sample presented lower socioeconomic status than the full cohort and with more Boys, which might have overestimated our PA estimates, despite no evidence of bias in the associations between PA and the potential determinants were found. Also, reverse causality could affect some of our associations, such as motor development and paternal PA which were collected at the same moment as infants’ PA. An additional limitation is that sleep time was included in the average daily acceleration, which may impact on the observed associations. A way of dealing with sleep time is to use a sleep diary, but this would be unfeasible in such a large sample and in situations where the child was in daycare. Also, sleep algorithms to differentiate sleep time from non-wear time and/or sedentary behavior are not available for this age group yet. Therefore, calibration studies are needed to develop such algorithms to enable the separation of sleep time and PA estimates. Finally, the magnitude of physical activity and the differences between group is challenging due to the absence of calibration studies in this specific age-group.

Our study has some strengths, such as the large population-based sample, detailed information on a number of potential determinants of PA, and the innovative use of accelerometry for assessing PA on such a young sample. Also, the use of raw accelerometry data analysis allows for transparency in all stages of data processing and higher comparability between data collected from different accelerometer brands. External validity of our findings depends on contextual similarities in terms of cultural and socioeconomic characteristics; however, we expect that the results presented might be extrapolated to one-year-old infants from middle-income settings.

## Conclusion

In conclusion, our findings demonstrate higher levels of PA among boys and those children with higher maternal PA during pregnancy, regardless of walking status. Also, among infants able to independently walk, intermediate groups of maternal schooling, adequate length-for-age (z-score), no daycare attendance and higher paternal PA are positive correlates of objectively measured PA early in life. These results could be useful to advise educational institutions, governments and parents regarding developmental and behavioral aspects of infants’ care.

## Data Availability

The data that support the findings of this study are available from Epidemiology Postgraduate Program but restrictions apply to the availability of these data, which were used under license for the current study, and so are not publicly available. Data are however available from the authors upon reasonable request and with permission of the Epidemiology Postgraduate Program Publication Committee.
